# Assessment of Boron Diffusivities in Nickel Borides by Two Mathematical Approaches

**DOI:** 10.3390/ma15020555

**Published:** 2022-01-12

**Authors:** Mourad Keddam, Peter Jurči

**Affiliations:** 1 Laboratoire de Technologie des Matériaux, Faculté de Génie Mécanique et Génie des Procédés, Université des Sciences et de la Technologie Houari-Boumediene, B.P N°32, El-Alia, Bab-Ezzouar, Algiers 16111, Algeria; keddam@yahoo.fr; 2 Department of Materials Science, Faculty of Material Sciences and Technology in Trnava, Slovak University of Technology, J. Bottu 25, 91724 Trnava, Slovakia

**Keywords:** boronizing, nickel borides, alternative diffusion model (ADM), MDC method, kinetics, activation energy

## Abstract

In the work of this contribution, two kinetics models have been employed to assess the boron diffusivities in nickel borides in case of Inconel 718 alloy. The first approach, named the alternative diffusion model (ADM), used the modified version of mass conservation equations for a three-phase system whilst the second one employed the mean diffusion coefficient (MDC) method. The boron diffusivities in nickel borides were firstly evaluated in the interval of 1123 to 1223 K for an upper boron concentration of 11.654 wt% in Ni_4_B_3_. The boron activation energies in the three phases (Ni_4_B_3_, Ni_2_B and Ni_3_B) were secondly deduced by fitting the values of boron diffusivities with Arrhenius relations. Finally, these values of energy were compared with the results from the literature for their experimental validation.

## 1. Introduction

The boronizing is a surface-hardening process used to harden the surfaces of ferrous alloys [1,2,3,4,5] and non-ferrous alloys [6,7,8,9,10,11,12] to get hard coatings on them. It is based on thermodiffusion of boron atoms through the surfaces of metallic substrates generally in the interval of 800–1050 °C for the holding times of 0.5–10 h [13]. This process brings about interesting surface properties such as high hardness, resistance to wear and corrosion for obtained boronized layers.

For hardening the metallic surfaces by boriding, many methods [6,8,11,12,14,15,16,17,18,19] can be employed to achieve this surface treatment. However, the solid boriding employing a mixture of powders is interesting because it is simpler and requires a low cost of investment in comparison with other variants of boriding processes [20].

The borided alloys [6,12,14,21] are used in many sectors of industry because of their intrinsitic functional properties. The domain of their utilization is very broad covering the following industries: marine, petrochemistry, aerospace and nuclear. As per the Ni-B binary system [22], the boride phases Ni_4_B_3_, NiB, Ni_2_B and Ni_3_B could be formed at equilibrium thermodynamic state.

To understand the phenomenon of boron diffusion at the surfaces of nickel-based alloys, it is necessary to develop kinetics approaches based on empirical [23,24] and mathematical models [6,12,24,25,26]. Thus, the aim of modeling the boron diffusion in these materials is to reduce the number of experiments by optimizing the thickness of boride coatings. Very little information is available in the literature regarding the modeling of the boriding kinetics of nickel-based alloys. In a recent study, Gunen et al. [6] employed the integral method to analyze the kinetics of boride coatings on Monel 400 alloy.

Contla-Pacheco et al. [12] also used the integral diffusion model for a three phase-system in case of borided Inconel 718 alloy to assess the boron diffusion coefficients in the three phases (Ni_4_B_3_, Ni_2_B and Ni_3_B). Recently, Makuch et al. [25] used the integral diffusion model for the boriding kinetics of Nimonic 80 A alloy treated by plasma paste boriding (PPB). In their study, the entire boronized layer composed of a mixture of nickel borides (Ni_4_B_3_, NiB, Ni_2_B and Ni_3_B) was considered having a mean value of thickness in the range 19.06–77.82 µm. Campos-Silva et al. [26] treated the Inconel 718 alloy with the pulsed-DC powder-pack boriding process between 1123 and 1223 K. They used the bilayer model applied to the dual–phase nickel boride layer and the transient zone for estimating the boron diffusivities in the (Ni_4_B_3_ + Ni_2_B) layer and diffusion zone.

In the contribution of this work, the alternative diffusion model (ADM) [27,28] and the mean diffusion coefficient (MDC) [29,30] applied to a system of three phases were suggested to assess the boron diffusivities in nickel borides. For the alternative diffusion model, expressions of boron diffusivities were obtained from the mass balance equations at three growth interfaces as a function of equilibrium boron concentrations and dimensionless parameters related to boride incubation periods. For the MDC method, the real experimental layers’ thicknesses were fitted with the parabolic relationships passing through the origin of time axis. Therefore, the extracted values of experimental kinetics constants were considered in the interval of 1123 to 1223 K for estimating the boron diffusivities in nickel borides. Finally, the boron activation energies in nickel borides were deduced and compared to those reported in the literature.

## 2. The two Diffusion Models

### 2.1. First Approach: The Alternative Diffusion Model

In this kinetic approach [27,28], the mass balance equations are formulated at the three interfaces within a saturated matrix with boron atoms. The local thermodynamic equilibrium prevailed during the saturation process of the material surface by interstitial atoms in a sufficient amount resulting in the formation of three layers. In the alternative diffusion model, the infinitesimal concentration of interstitial element $dC_{i}(x,t)$ within each layer, after a certain time t and diffusion distance x, is given by Equation (1) for a selected process temperature:

(1) $$dC_{i}(x,t)=\frac{\partial C_{i}(x,t)}{\partial t}dt+\frac{\partial C_{i}(x,t)}{\partial x}dx$$

The partial derivative of $dC_{i}(x,t)$ (i = 1 to 3) with respect to the diffusion distance x [27,28] is related to its exact differential by the following formula (see Equation (2)):

(2) $$\frac{\partial C_{{}_{I}}(x,t)}{\partial x}=\frac{1}{2}\frac{dC_{i}(x,t)}{dt}\frac{dt}{dx}$$

**Figure 1:**
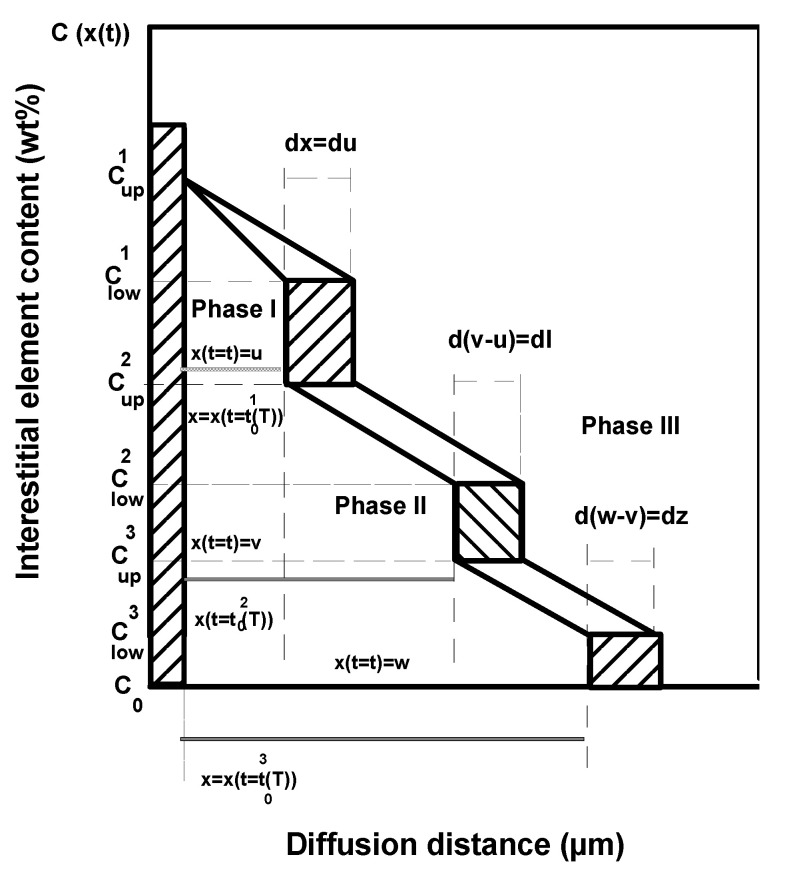
Schematic representation of interstitial element concentration profile inside a system of three phases.

Figure 1 shows the distribution of interstitial element within a three-phase system. The upper and lower concentrations of interstitial element are, respectively, $C_{up}^{i}$ and $C_{low}^{i}$ in the ith layer. The variable u(t) is the location of the first interface (I/II). The variable v(t) represents the location of the second interface (II/III) whilst the variable w(t) is relative to the third interface. $C_{0}$ is the solubility of interstitial element in the substrate. The mass balance equation describing the motion of the first interface as a function of treatment time at a distance u(t) from the material surface is given by Equation (3):

(3) $$w_{1}{\frac{dx}{dt}|}_{x=u}=-D_{1}{\frac{\partial C_{1}}{\partial x}|}_{x=u}+D_{2}{\frac{\partial C_{2}}{\partial x}|}_{x=u}$$

Considering the principle of mass conservation, the mass balance equation at the second interface located at x=v(t) is given by Equation (4):

(4) $$w_{2}{\frac{dx}{dt}|}_{x=v}+w_{12}{\frac{dx}{dt}|}_{x=u}=-D_{2}{\frac{\partial C_{2}}{\partial x}|}_{x=v}+D_{3}{\frac{\partial C_{3}}{\partial x}|}_{x=v}$$

For the third interface, the mass balance equation at the position x=w(t) is expressed by:

(5) $$w_{3}{\frac{dx}{dt}|}_{x=w}+w_{23}{\frac{dx}{dt}|}_{x=v}=-{D_{3}\frac{\partial C_{3}}{\partial x}|}_{x=w}$$

with

$$w_{1}=0.5(C_{up}^{1}-C_{low}^{1})+(C_{low}^{1}-C_{up}^{2}),\;w_{2}=0.5(C_{up}^{2}-C_{low}^{2})+(C_{low}^{2}-C_{up}^{3}),\;\;w_{3}=0.5(C_{up}^{3}-C_{low}^{3})+(C_{low}^{3}-C_{0}),w_{12}=0.5(C_{up}^{2}-C_{low}^{2}),\;\;w_{23}=0.5(C_{up}^{3}-C_{low}^{3}),$$

The parameter $D_{i}$ with (i = 1 to 3) represents the diffusivity of the interstitial element in the phase i = 1, 2 or 3.

The thickness of layer I u(t) is expressed by Equation (6):

(6) $$u(t)=k_{1}{\left[t-t_{0}^{1}(T)\right]}^{0.5}$$

where $k_{1}$ is the parabolic growth constant at the (I/II) interface for a boride incubation time $t_{0}^{1}(T)$. The layer thickness of (I + II) v(t) is provided by Equation (7):

(7) $$v(t)=k_{2}{\left[t-t_{0}^{2}(T)\right]}^{0.5}$$

where $k_{2}$ is the parabolic growth constant at the (II/III) interface for a boride incubation time $t_{0}^{2}(T)$.The layer thickness of (I + II + III) w(t) is given by Equation (8):

(8) $$w(t)=k_{3}{\left[t-t_{0}^{3}(T)\right]}^{0.5}$$

where $k_{3}$ is the parabolic growth constant at the (III/substrate) interface for a boride incubation time $t_{0}^{3}(T)$. Mathematically, Equations (6)–(8) can be re-written as follows:

(9) $$u(t)=\lambda_{1}\sqrt{t}$$

(10) $$v(t)=\lambda_{2}\sqrt{t}$$

and

(11) $$w(t)=\lambda_{3}\sqrt{t}$$

where the incubation times are virtually set to zero, the constants λ_i_ (i = 1 to 3) are the new values of kinetics constants at three interfaces obtained from the kinetics curves of experimental data. Equations (12)–(14) were obtained, by considering the relation (2), the time derivatives of Equations (9)–(11) as well as the integration of both sides of Equations (3)–(5):

(12) $$w_{1}{\int_{t_{0}^{1}}^{t}\left(\frac{du}{dt}\right)}^{2}dt=-\frac{1}{2}\int_{C_{up}^{1}}^{C_{low}^{1}}D_{1}dC_{1}+\frac{1}{2}\int_{C_{up}^{2}}^{C_{low}^{2}}D_{2}dC_{2}$$

(13) $$\int_{t_{0}^{2}}^{t}(w_{2}({\frac{dx}{dt}{)}^{2}|}_{x=v}+w_{12}{\frac{dx}{dt}|}_{x=u}{\frac{dx}{dt}|}_{x=v})dt=-\frac{1}{2}\int_{C_{up}^{2}}^{C_{low}^{2}}D_{2}dC_{2}+\frac{1}{2}\int_{C_{up}^{3}}^{C_{low}^{3}}D_{3}dC_{3}$$

(14) $$\int_{t_{0}^{3}}^{t}(w_{3}({\frac{dx}{dt}{)}^{2}|}_{x=w}+w_{23}{\frac{dx}{dt}|}_{x=v}{\frac{dx}{dt}|}_{x=w})dt=-\frac{1}{2}\int_{C_{up}^{3}}^{C_{low}^{3}}D_{3}dC_{3}$$

Equations (12)–(14) can be also rewritten as follows:

(15) $$w_{1}\int_{t_{0}^{1}}^{t}\frac{\lambda_{1}^{2}}{4t}dt=\frac{w_{1}\lambda_{1}^{2}}{4}\ln(\frac{t}{t_{0}^{1}})=-\frac{1}{2}\int_{C_{up}^{1}}^{C_{low}^{1}}D_{1}dC_{1}+\frac{1}{2}\int_{C_{up}^{2}}^{C_{low}^{2}}D_{2}dC_{2}$$

(16) $$\begin{array}{l} w_{2}\int_{t_{0}^{2}}^{t}\frac{\lambda_{2}^{2}}{4t}dt+\int_{t_{0}^{2}}^{t}\frac{w_{12}\lambda_{1}\lambda_{2}}{4t}dt=-\frac{1}{2}\int_{C_{up}^{2}}^{C_{low}^{2}}D_{2}dC_{2}+\frac{1}{2}\int_{C_{up}^{3}}^{C_{low}^{3}}D_{3}dC_{3} \end{array}$$

(17) $$w_{3}\int_{t_{0}^{3}}^{t}\frac{\lambda_{3}^{2}}{4t}dt+\int_{t_{0}^{3}}^{t}\frac{w_{23}\lambda_{2}\lambda_{3}}{4t}dt=-\frac{1}{2}\int_{C_{up}^{3}}^{C_{low}^{3}}D_{3}dC_{3}$$

After rearrangement and some mathematical manipulations in the above expressions, Equations (18)–(20) were then obtained:

(18) $$\frac{1}{2}w_{1}\lambda_{1}^{2}\ln(\frac{t}{t_{0}^{1}})=D_{1}(C_{up}^{1}-C_{low}^{1})-D_{2}(C_{up}^{2}-C_{low}^{2})$$

(19) $$\frac{1}{2}(w_{2}\lambda_{2}^{2}+w_{12}\lambda_{1}\lambda_{2})\ln(\frac{t}{t_{0}^{2}})=D_{2}(C_{up}^{2}-C_{low}^{2})-D_{3}(C_{up}^{3}-C_{low}^{3})$$

(20) $$\frac{1}{2}(w_{3}\lambda_{3}^{2}+w_{23}\lambda_{2}\lambda_{3})\ln(\frac{t}{t_{0}^{3}})=D_{3}(C_{up}^{3}-C_{low}^{3})$$

Finally, the expressions of diffusivities of interstitial element in the three phases were given by Equations (21)–(23):

(21) $$D_{1}=\frac{\left[w_{1}\lambda_{1}^{2}\ln(\frac{t}{t_{0}^{1}})+(w_{2}\lambda_{2}^{2}+w_{12}\lambda_{1}\lambda_{2})\ln(\frac{t}{t_{0}^{2}})+(w_{3}\lambda_{3}^{2}+w_{23}\lambda_{2}\lambda_{3})\ln(\frac{t}{t_{0}^{3}})\right]}{2(C_{up}^{1}-C_{low}^{1})}$$

(22) $$D_{2}=\frac{\left[(w_{2}\lambda_{2}^{2}+w_{12}\lambda_{1}\lambda_{2})\ln(\frac{t}{t_{0}^{2}})+(w_{3}\lambda_{3}^{2}+w_{23}\lambda_{2}\lambda_{3})\ln(\frac{t}{t_{0}^{3}})\right]}{2(C_{up}^{2}-C_{low}^{2})}$$

(23) $$D_{3}=\frac{\left(w_{3}\lambda_{3}^{2}+w_{23}\lambda_{2}\lambda_{3})\ln(\frac{t}{t_{0}^{3}}\right)}{2(C_{up}^{3}-C_{low}^{3})}$$

It is noteworthy to explain the definition of three temperature-dependent parameters Φ_i_, (i = 1 to 3) which are in relation with the $\frac{t}{t_{0}^{i}(T)}$ ratios. These parameters were deduced from the above equalities in terms of layers’ thicknesses:

(24) $$\lambda_{i}\sqrt{t}=k_{i}\sqrt{t-t_{0}^{i}(T)}=k_{i}\sqrt{t}\sqrt{1-\frac{t_{0}^{i}(T)}{t}}=k_{i}\sqrt{t}\Phi_{i}$$

where $\Phi_{i}=\sqrt{1-\frac{t_{0}^{i}(T)}{t}}=\frac{\lambda_{i}}{k_{i}}$

### 2.2. Second Approach: The Mean Diffusion Coefficient Method

In this kinetic approach [29,30], the profile of interstitial element concentration is assumed to be linear in each layer of thickness $\Delta x_{i}$ (i = 1 to 3) within a three-phase system. Therefore, the diffusivity of interstitial element within each layer can be expressed by Equation (25):

(25) $$D_{i}=\frac{\Delta x_{i}(\sum_{j=1}^{j=3}\gamma_{ij}\Delta x_{j})}{2t\Delta C_{i}}$$

For this three-phase system, the thickness of each layer is the following:

$$\Delta x_{1}=u=\lambda_{1}\sqrt{t},\;\Delta x_{2}=(v-u)=(\lambda_{2}-\lambda_{1})\sqrt{t},\Delta x_{3}=(w-v)=(\lambda_{3}-\lambda_{2})\sqrt{t}$$

with $\gamma_{ii}=0.375C_{up}^{i}+0.625C_{low}^{i}$ for i = 1 to 3,

$$\gamma_{12}=\gamma_{21}=0.5(C_{up}^{2}+C_{low}^{2}),\;\gamma_{13}=\gamma_{31}=\gamma_{23}=\gamma_{32}=0.5(C_{up}^{3}+C_{low}^{3})$$

## 3. Simulation Results and Discussions

The experimental data published by Contla-Pacheco et al. [12] have been exploited with the aim of assessing the boron diffusivities in nickel borides based on the two approaches (the alternative diffusion model and the mean diffusion coefficient method) for Inconel 718 alloy. In their experimental study [12], the boronizing process was realized in an electrical muffle furnace without any protective atmosphere on the substrates of Inconel 718 alloy containing in (weight percent): 50–55% Ni (+Co), 17–21% Cr, 4.75–5.25% Nb (+Ta), 2.80–3.30% Mo, 0.65–1.15% Ti, 0.20–0.80% Al, 0.085% C, 1.00% Co max., 0.05% C max., 0.35% Mn max., 0.35% Si max., 0.015% P max., 0.002% S max., 0.006% B max., 0.30% Cu max., 0.01% N max. and Fe balance. The process parameters were 1123, 1173 and 1223 K for 2, 4 and 6 h. The powders mixture had the following chemical composition (in weight percent): 90% B_4_C and 10% KBF_4_. The samples to be treated were wrapped in the powder mixture and placed in a container made of AISI 304 stainless steel. The XRD analysis identified the presence of nickel boride phases (Ni_4_B_3_, Ni_2_B and Ni_3_B). In addition, the cross-sectional views of boronized specimens showed three distinct zones when examining their microstructures by scanning electron microscope [12]. For kinetic studies, the measurements of layers were made on different locations of the cross-sections of boronized samples. The experimental kinetics constants at the three growing interfaces: (Ni_4_B_3_/Ni_2_B), (Ni_2_B/Ni_3_B) and (Ni_3_B/substrate) with the associated boride incubation periods were taken from [12].

Such experimental values [12] were deduced from the slopes of the straight lines describing the time dependencies of $u^{2}$, $v^{2}$ and $w^{2}$ according to Equations (6)–(8). The determined boride incubation times correspond to the intercepts with time axis in the plots. It is seen from the reference work [12] that the boride incubation times are decreased with increasing process temperatures [12,15,16,19,25] (as experienced in other studies) due to thermal activated process of boron diffusion. In Table 1 are listed the new values of kinetics constants relative to three growth interfaces fitted with Equations (9)–(11).

Table 2 gives the calculated values of Φ_1_, Φ_2_, Φ_3_ parameters versus the boriding temperature. It is clear that these three parameters do not change significantly with the process temperature. Therefore, a mean arithmetic value for each parameter was considered as in previous studies for either one phase [27] or bilayer system [28]. Such an approximation allows us to assess the boron diffusivities in nickel borides.

### 3.1. Estimation of Boron Diffusivities in Nickel Borides

In order to make the necessary calculations about the boron diffusivities in nickel borides, the values of upper and lower boron concentrations in each phase are needed. Based on the reported values of boron concentrations [12], $C_{up}^{1}$ and $C_{low}^{1}$ are, respectively, equal to 11.615 and 11.50 wt% B for the Ni_4_B_3_ phase. $C_{up}^{2}$ and $C_{low}^{2}$ have the following values (11.615 and 11.50 wt% B) for the Ni_2_B phase. For the Ni_3_B phase, the maximum and minimum boron contents are, respectively, $C_{up}^{3}$ = 6.17 wt% and $C_{low}^{3}$ = 6 wt% B. Based on the data of Table 2, the mean values taken for the Φ_1_, Φ_2_, Φ_3_ parameters are the following: 0.895, 0.924 and 0.9553 for estimating the boron diffusivities in nickel borides.

Table 3 provides the calculated values of diffusion coefficients of boron in nickel borides with Equations (21)–(23) for the three temperatures 1123, 1173 and 1223 K with a value of maximum boron content in Ni_4_B_3_ of 11.615 wt%.

**Figure 2:**
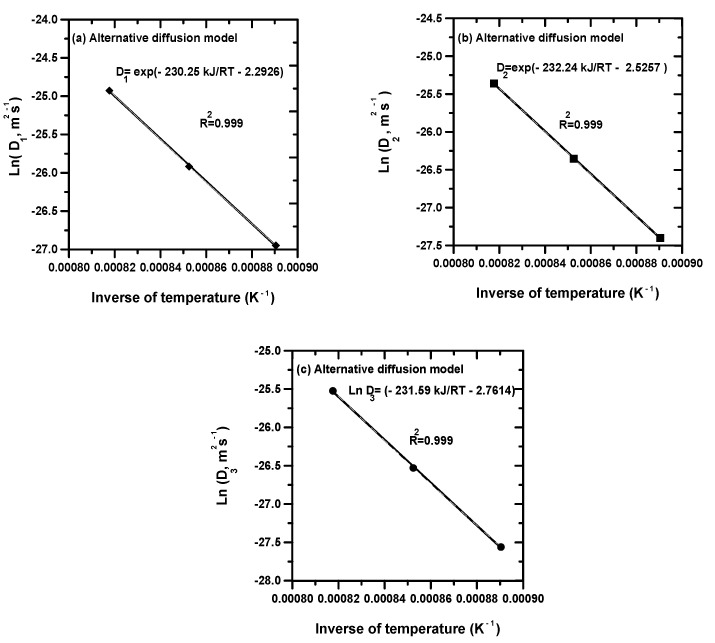
Arrhenius behaviors of assessed boron diffusivities in nickels borides with the alternative diffusion model.

Figure 2 gives the temperature dependence of calculated boron diffusivities in nickel borides with the use of alternative diffusion model in the interval of 1123 to 1223 K. By adopting Arrhenius relationships, the following expressions for the temperature dependence of boron diffusion coefficients in each boride layer (in m^2^ s^−1^) were obtained:

(26) $$D_{1}=1.01\times 10^{-1}\exp(\frac{-230.25kJ/mol}{RT})$$

(27) $$D_{2}=8\times 10^{-2}\exp(\frac{-232.24kJ/mol}{RT})$$

(28) $$D_{3}=6.32\times 10^{-2}\exp(\frac{-231.59kJ/mol}{RT})$$

where: *T*—the absolute temperature (K), *R*—the ideal gas constant (*R* = 8.314 J mol^−1^·K^−1^).

Table 4 contains the estimated values of boron diffusion coefficients in nickel borides with the mean diffusion coefficient (MDC) method.

**Figure 3:**
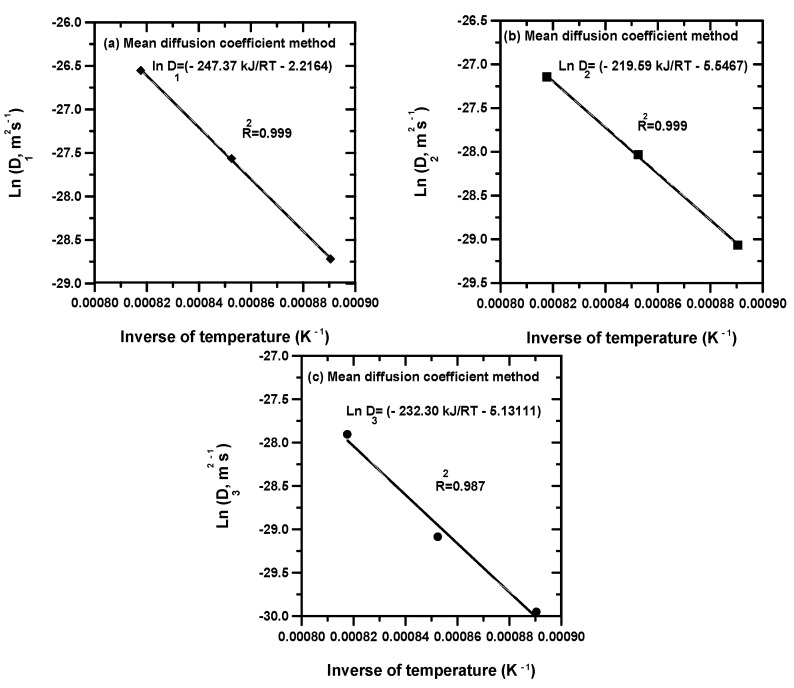
Arrhenius behaviors of calculated boron diffusivities in nickels borides with the help of MDC method.

In Figure 3 are plotted the fitted values of Table 4 with Arrhenius relationships based on the MDC method. The results of this regression were given by Equations (29)–(31):

(29) $$D_{1}=1.09\times 10^{-1}\exp(\frac{-247.37kJ/mol}{RT})$$

(30) $$D_{2}=3.9\times 10^{-3}\exp(\frac{-219.59kJ/mol}{RT})$$

(31) $$D_{3}=5.91\times 10^{-3}\exp(\frac{-232.30kJ/mol}{RT})$$

### 3.2. Comparing the Values of Boron Activation with Those Found in the Literature

For both approaches, the values of boron activation energies in nickel borides can be deduced from the slopes of straight lines displayed in Figure 2 and Figure 3. Table 5 shows a comparison of boron activation energies in nickel borides obtained on some nickel alloys and nickel aluminide substrates [6,12,23,24,25,26,31,32] along with the present results. It is noted that the reported values of boron activation energies in nickel borides depended on the boriding method, the temperature interval selected, the chemical composition of base material and the calculation method. For information, Gunen et al. [6] treated the surfaces of Monel 400 alloy with the powder method (using a powder mixture of 90 wt% B_4_C and 10 wt% NaBF_4_) to produce the Ni_2_B layer between 1173 and 1273 K. The associated value of activation energy in this case was 300.9 kJ mol^−1^. In another study, Campos-Silva et al. [26] used a novel method for boriding named pulsed-DC powder-pack boriding process (PDCPB) to generate boronized layers on Inconel 718 alloy. In this process, the value of current supply was set to 5 A using cycles of inversion polarity of 10 s. Thereby, the pulsed direct current assures a uniform diffusion of boron atoms in both surfaces (of anode and cathode). Therefore, it accelerates the diffusion phenomenon of boron compared to the conventional powder method [6,12,23,26]. They applied a diffusion model [26] for assessing the boron diffusion coefficients through the bilayer (Ni_4_B_3_ + Ni_2_B) and inside the diffusion zone. The obtained activation energies (153 kJ mol^−1^ for the bilayer and 159 kJ mol^−1^ for the diffusion zone) [26] are lower compared to the results of the conventional powder method due to the influence of electromigration during the diffusion of boron. In reference [12], the activation energies for boron diffusion in the three phases (Ni_4_B_3_, Ni_2_B and Ni_3_B) have been determined by the integral method when employing the powder-pack boriding process [12]. The obtained values are quite higher compared to the PDCPB ascribed to the activation of mass transport. Makuch et al. [25] used the integral method to investigate the boron diffusion in a multiphase system consisting of a mixture of nickel borides when boronizing the Nimonic 80A alloy with the plasma paste boriding (PPB). The assessed value of activation energy in this system was 190.93 kJ mol^−1^. Kahvecioglu et al. [31] carried out an ultra-fast electrochemical boriding on the nickel aluminide substrates in the temperature range 1073–1223 and by varying the values of current density between 0.1 and 0.5 Acm^−2^. The estimated value of boron activation in this material was 185.96 kJ mol^−1^. In addition, Torun [32] performed the boriding process on the Ni_3_Al alloy with Ekabor-Ni powders in the temperature range 1073–1223 K between 2 and 8 h. In this work, the value of activation energy for boron diffusion in the nickel aluminide substrate was calculated as 118.8 ± 14.4 kJ mol^−1^ with a boride layer composed of Ni_3_B and Ni_4_B_3_ phases. It is seen that the assessed values of boron activation energies in nickel borides from both approaches for Inconel 718 alloy are concordant with the literature data [6,12,23,25]. The obtained results in terms of activation energies allowed us to validate the two diffusion models.

## 4. Conclusions

In this study, two kinetics approaches have been suggested to obtain the values of boron diffusion coefficients in nickel borides in case of Inconel 718 alloy. In the alternative diffusion model, expressions of boron diffusivities were derived from the mass balance equations at three growth interfaces as a function of equilibrium boron concentrations and dimensionless parameters related to boride incubation periods. In the mean diffusion coefficient method, the corresponding expressions for boron diffusion coefficients in nickel borides are depending on both the equilibrium boron concentrations and the kinetics constants at three growth interfaces by adopting a linear boron concentration profile in each individual phase. For the alternative diffusion model, the boron activation energies in Ni_4_B_3_, Ni_2_B and Ni_3_B were, respectively, 230.25, 232.24 and 231.59 kJ mol^−1^. For the MDC method, the Ni_4_B_3_, Ni_2_B and Ni_3_B layers had the respective boron activation energies: 247.37, 219.59 and 232.30 kJ mol^−1^. Furthermore, the values of activation energies in the three phases (Ni_4_B_3_, Ni_2_B and Ni_3_B) from both approaches are consistent with the data reported in the literature. In future works, the two models can be exploited to understand the diffusion phenomenon of interstitial elements in a multiphase system forming individual compact layers.

## Figures and Tables

**Table 1:** New values of experimental kinetics constants fitted with Equations (9)–(11).

T (K)	$\lambda_{1}\;(\times 10^{-8}\;m\;s^{-0.5})$ at the First Interface	$\lambda_{2}\;(\times 10^{-8}\;m\;s^{-0.5})$ at the Second Interface	$\lambda_{3}\;(\times 10^{-8}\;m\;s^{-0.5})$ at the Third Interface
1123	5.51	12.06	15.60
1173	10.21	21.10	26.10
1223	17.12	33.31	43.15

**Table 2:** Calculated values of dimensionless parameters Φ_i_ with i = (1 to 3) based on Equation (24).

T (K)	$\Phi_{1}\;\mathrm{Parameter}$	$\Phi_{2}\;\mathrm{Parameter}$	$\Phi_{3}\;\mathrm{Parameter}$
1123	0.8830	0.9090	0.9190
1173	0.8920	0.9320	0.9690
1223	0.9110	0.9330	0.9780

**Table 3:** Estimated values of boron diffusivities in nickel borides by the alternative diffusion model.

T (K)	*D*_1_ $\left(\times 10^{-12}\;m^{2}\;s^{-1}\right)$	*D*_2_ $\left(\times 10^{-12}\;m^{2}\;s^{-1}\right)$	*D*_3_ $\left(\times 10^{-12}\;m^{2}\;s^{-1}\right)$
1123	1.98	1.26	1.07
1173	5.54	3.59	3.00
1223	14.93	9.69	8.20

**Table 4:** Estimated values of boron diffusivities in nickel borides by the MDC method.

T (K)	*D*_1_ $\left(\times 10^{-13}\;m^{2}\;s^{-1}\right)$	*D*_2_ $\left(\times 10^{-13}\;m^{2}\;s^{-1}\right)$	*D*_3_ $\left(\times 10^{-13}\;m^{2}\;s^{-1}\right)$
1123	3.37	2.38	0.98
1173	10.68	6.70	2.33
1223	29.39	16.29	7.59

**Table 5:** Values of boron activation energies in nickel alloys for different boronizing processes.

Alloy	Boriding Process	Temperature Range (K)	Phases of the Boronized Layer	Activation Energies (kJ mol^−1^)	Method Used	Refs.
Monel 400	Powder	1123–1273	Ni_2_B	300.7	Integral method	[6]
Inconel 718	Powder	1123–1223	Cr_2_B, Ni_2_B, Ni_3_B, Ni_4_B_3_	233.20 (Ni_4_B_3_) 206.17 (Ni_2_B) 218.06 (Ni_3_B)	Integral method	[12]
Nickel 201	Powder	1123–1223	NiB, Ni_2_B, Ni_3_B, Ni_4_B_3_	203.87	Empirical relation	[23]
Ni-Mg at 3 and 7 wt% Mg	Powder	1173–1273	NiB, Ni_2_B, Ni_3_B	58.843 for 3 wt% Mg 136.506 for 7 wt% Mg	Empirical relation	[24]
Nimonic 80 A	Plasma paste Boriding	1023–1123	NiB, Ni_2_B, Ni_3_B, Ni_4_B_3_	190.93	Integral method	[25]
Inconel 718	Pulsed-DC powder	1123–1223	Ni_4_B_3_, Ni_2_B, Fe_2_B, Cr_2_B	153 for the bilayer 159 for the diffusion zone	Bilayer model	[26]
Ni_3_Al	Electrochemical	1073–1223	Ni_3_B, Ni_4_B_3_, Ni_20_AlB_14_	185.95	Empirical relation	[31]
Ni_3_Al	Powder	1073–1223	Ni_3_B, Ni_4_B_3_, Ni_3_Al	188 ± 14.4	Empirical relation	[32]
Inconel 718	Powder	1123–1223	Ni_2_B, Ni_3_B, Ni_4_B_3_	230.25 (Ni_4_B_3_) 232.24 (Ni_2_B) 231.59 (Ni_3_B)	Alternative diffusion model	This work
				247.37 (Ni_4_B_3_) 219.59 (Ni_2_B) 232.30 (Ni_3_B)	MDC method	

## Data Availability

The authors confirm that the data supporting the findings of this study is available within the article.
